# 1400 W, a selective inducible nitric oxide synthase inhibitor, mitigates early neuroinflammation and nitrooxidative stress in diisopropylfluorophosphate-induced short-term neurotoxicity rat model

**DOI:** 10.3389/fnmol.2023.1125934

**Published:** 2023-03-17

**Authors:** Nyzil Massey, Suraj Sundara Vasanthi, Manikandan Samidurai, Meghan Gage, Nikhil Rao, Christina Meyer, Thimmasettappa Thippeswamy

**Affiliations:** Biomedical Sciences, Iowa State University, Ames, IA, United States

**Keywords:** organophosphate nerve agent, 1400W, neuroinflammation, neurodegeneration, iNOS, epilepsy, cytokines, nitrooxidative stress

## Abstract

Organophosphate nerve agent (OPNA) exposure induces acute and long-term neurological deficits. OPNA exposure at sub-lethal concentrations induces irreversible inhibition of acetylcholinesterase and cholinergic toxidrome and develops *status epilepticus* (SE). Persistent seizures have been associated with increased production of ROS/RNS, neuroinflammation, and neurodegeneration. A total of 1400W is a novel small molecule, which irreversibly inhibits inducible nitric oxide synthase (iNOS) and has been shown to effectively reduce ROS/RNS generation. In this study, we investigated the effects of 1400W treatment for a week or two weeks at 10 mg/kg or 15 mg/kg per day in the rat diisopropylfluorophosphate (DFP) model. 1400W significantly reduced the number of microglia, astroglia, and NeuN+FJB positive cells compared to the vehicle in different regions of the brain. 1400W also significantly reduced nitrooxidative stress markers and proinflammatory cytokines in the serum. However, neither of the two concentrations of 1400W for two weeks of treatment had any significant effect on epileptiform spike rate and spontaneous seizures during the treatment period in mixed sex cohorts, males, or females. No significant sex differences were found in response to DFP exposure or 1400W treatment. In conclusion, 1400W treatment at 15 mg/kg per day for two weeks was more effective in significantly reducing DFP-induced nitrooxidative stress, neuroinflammatory and neurodegenerative changes.

## 1. Introduction

Chemical warfare agents pose a threat to humans. Among them, neurotoxic agents such as organophosphates (OP) are very important due to their fatality, long-term health effects, and hazard. Historically OP have been used as insecticides, nerve agents (OPNA) and even in medications (King and Aaron, 2015). OPNA exposure also remain as a threat to farming community in Africa and South East Asian countries (Peter and John, 2008). The exposure to OPNA can occur from drinking, breathing or through skin contact. It has been estimated that close to 3 million people suffer from OP poisoning worldwide which results in approximately two hundred thousand deaths (Wilkins, 2017). The use of pesticide in US is heavily regulated under the Federal Insecticide, Fungicide and Rodenticide Act (FIFRA) (2020). Aside from its hazard to the farming community, a major threat from OPNA as a warfare agent is a very realistic possibility to civilian and military personnel (Jett, 2010, 2012; Jett et al., 2020). OPNA like Sarin, soman, GA, VX, soman and Novichok are some of the agents that can be used in warfare (Yanagisawa et al., 2006; Dolgin, 2013; Kloske and Witkiewicz, 2019). These OPNAs, depending on the concentrations, can have acute, chronic and delayed symptoms.

Typical acute symptoms of OP exposure include muscle weakness, fasciculation, tremors, convulsions, muscle cramps, paralysis, and death (Bakry et al., 1988; Shih et al., 1991; McDonough and Shih, 1997; Reddy and Colman, 2017). The major concerns of acute OP exposure are convulsive seizures and *status epilepticus* (SE), which when lasts for more than 20 min can result in death or neuronal damage and behavioral dysfunction in survivors (Chen, 2012; Hobson et al., 2018; Scholl et al., 2018). OPNA irreversibly inhibits the acetylcholinesterase (AChE), which leads to acetylcholine (ACh) build up at the synaptic clefts and over stimulation of ACh receptors. Excess stimulation of muscarinic ACh receptors causes visual impairment, bronchoconstriction, increased salivation, lacrimation, and urination. Whereas, nicotinic ACh receptors activation in the CNS induces convulsions, ataxia, and seizures (Eskenazi et al., 1999; Leibson and Lifshitz, 2008). Exposure to OPNA in rats has also been known to induce significant nitrooxidative stress, cognitive decline, and epilepsy (Andersen, 2004; Furtado et al., 2010; Wright et al., 2010; Puttachary et al., 2015; Rowley et al., 2015; Flannery et al., 2016). Brain is sensitive to free radicals and has ~10 times lower antioxidant activity than other organs (Mendez-Armenta et al., 2014). Basal level reactive oxygen species (ROS) and reactive nitrogen species (RNS) are required for housekeeping functions such as autophagy, chemical signaling, cell division, and apoptosis (Mao and Franke, 2013). However, excessive free radicals (ROS/RNS) production if unregulated can impact cell function by modulating the structural proteins, enzymes, and DNA components (Cardenas-Rodriguez et al., 2013). RNS is produced by the combination of a second messenger molecule, nitric oxide (NO) and superoxide radicals. The different forms of RNS produced by NO include; nitric oxide radical (NO•), peroxynitrite radical (ONOO−), nitroxyl anion HNO−, nitrosonium cation (NO+), higher oxides of nitrogen (N2O3, NO2 •), and S-nitrosothiols (RSNO) (Cheeseman, 1993; Halliwell et al., 1999; Martinez and Andriantsitohaina, 2009). NO synthase (NOS) catalyzes the production of NO from the substrate L-arginine. NOS exists in 3 isoforms: a) neuronal NOS (nNOS) produced by neurons and its role in trauma/seizure-induced response can be transient (Cosgrave et al., 2008; Beamer et al., 2012), (b) endothelial NOS (eNOS) expressed mainly by the endothelial cells, and (c) inducible NOS (iNOS) produced in immune cells, astrocytes, microglia, and also neurons as a biproduct of inflammation in response to infection or trauma. Therefore, NO production mediated by iNOS in response to brain injury such as OPNA-induced SE is a potential target for disease modification in epilepsy.

There are proven medical countermeasures (MCM) for acute OP intoxication (Eddleston et al., 2008).The current MCM for OP toxicity includes atropine, pralidoxime (2-PAM), and diazepam or midazolam. However, they are effective in controlling only the acute symptoms of OP and none of them have long-term protective properties (McDonough and Shih, 1997; Eddleston et al., 2008; McDonough et al., 2010). The role of oximes is not well established and they might only work against specific pesticide poisoning with poor brain permeability (Eddleston et al., 2008). Diazepam/midazolam is a benzodiazepine anticonvulsant agent and can be useful if given within 20 min of OP exposure (Gilbert et al., 1999; McDonough et al., 1999; Shih et al., 1999; Capacio et al., 2004; Reddy and Reddy, 2015; Smith and Brown, 2017). However, recurrence and resistance to benzodiazepines following initial protection is a significant problem in OPNA-induced long-term neurotoxicity (McDonough et al., 1999; Leikin et al., 2002; Chapman et al., 2015). Therefore, our hypothesis was that a combination of MCM and an antioxidant such as a highly selective iNOS inhibitor, 1400W (Garvey et al., 1997) will mitigate diisopropylfluorophosphate (DFP)-induced brain injury. DFP is considered a surrogate of OPNA (Heiss et al., 2016; Chaubey et al., 2019).

Brain pathology and mitigation by interventional drugs depend on the initial SE severity and the duration of treatment. Previously, we had demonstrated the efficacy of 1400W at 20 mg/kg (i.m) in a rat DFP model that had mild to moderate SE. The treatment duration was only for the first 3 days and twice daily post-OPNA exposure. Since high concentrations of the drug can be toxic for long term treatment, in this study we tested two lower concentrations (10 and 15 mg/kg per day) for a week or 2 weeks to target all severity groups. We investigated the effects of these treatment regimens on gliosis, neurodegeneration, RNS/ROS markers, cytokines, and electroencephalogram (EEG) parameters.

## 2. Materials and methods

### 2.1. Animal studies and ethics statement

Young adult male and female Sprague Dawley rats (8 weeks old) were used in this study. The animals were procured from Charles River (Wilmington, MA, United States) and maintained in the Laboratory of Animal Resources at the Iowa State University. Animals were single housed in a controlled environment (19–23°C, 12 h light: 12 h dark), with *ad libitum* access to food and water. After 3 days of acclimatization, the animals were randomized and used in the experiments. All experiments were conducted in accordance with the Institutional Animal Care and Use Committee as per the approved protocols (IACUC-21-109 and 21–110). Aseptic techniques were followed for all surgical procedures, where proper pre- and post-operative care was provided with daily observation and bodyweight monitoring. Similar care was also provided to animals after DFP exposure. At the end of each experiment, all animals were euthanized with 100 mg/kg pentobarbital sodium (i.p.) as per the American Veterinary Medical Associations Guidelines for the Euthanasia.

### 2.2. Chemicals and reagents

All key chemicals used in this study were authenticated by LC/GC–MS/MS and the chemicals purity and identity were determined before testing in animals. DFP (Sigma-Aldrich, purity, 97.8% by GC–MS) was prepared fresh in ice cold PBS on the day of administration. Atropine sulfate (ATS, 99.9% pure by LC–MS, Tokyo Chemical Industry (TCI), United States) and 2-pralidoxime (2-PAM, 99.4% pure by LC–MS, Sigma) were also prepared fresh in saline on the day of administration. Midazolam (MDZ) was procured from the Pharmacy at ISU Lloyd Veterinary Medical Center Hospital. 1400W (≥ 99% pure- by HPLC, Tocris Bioscience, Bristol, England) was diluted in distilled water at 20 mg/ml. DFP exposure, treatment, and experimental groups.

### 2.3. Diisopropylfluorophosphate exposure, treatment, and experimental groups

**Figure 1:**
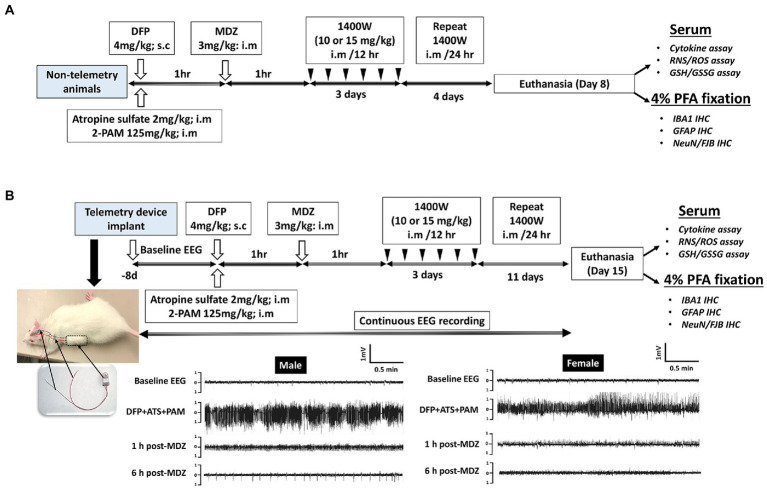
Experimental design for non-telemetry **(A)** and telemetry groups **(B)** illustrating the DFP exposure, intervention, and the endpoints.

A total of 86 animals were used in this study. A total of 34 animals were used for telemetry and rest without telemetry (denoted as “non-telemetry animals” in this study). Each experimental group had male and female rats (Table 1). The animals were randomized, grouped, and coded before they were used in the experiments. The experimental design is illustrated in Figure 1. Animals were either administered with DFP (4 mg/kg, s.c.) or vehicle and immediately (within a minute) followed by atropine sulfate (2 mg/kg, i.m.) and 2-PAM (25 mg/kg, i.m.) injections to control the peripheral effects of AChE inhibition. Following DFP injection, behavioral SE severity was scored using a modified Racine scale for 60 min. Midazolam (MDZ, 3 mg/kg, i.m.) was administered 1 h post-DFP to limit mortality and to control behavioral seizures. Animals in DFP exposed group were then randomly allotted to experimental groups with matched SE severity (Table 1). One group was treated with the vehicle and the other with 1400W (10 mg/kg or 15 mg/kg for 7 or 14 days). Some animals were also treated with 1400W alone, without DFP, as an additional control. All animals were perfused with 4% paraformaldehyde under terminal anesthesia at the end of the experiment. Brains were isolated and processed for immunohistochemistry to quantify the markers of neurodegeneration and gliosis. The serum was used for nitrooxidative and cytokine assays.

**Table 1:** Animal numbers in different groups and mean SE severity.

**Group**	**Sex**	**Vehicle**	**Vehicle + 1400W**	**DFP+ vehicle**	**DFP+ 1400W (10 mg/kg)**	**DFP+ 1400W (15 mg/kg)**
Non-telemetry	Males	4	4	6	4	5
	Females	4	4	5	5	6
	Total	8	8	11	9	11
Telemetry	Males	4	No animals	4	5	6
	Females	5	No animals	5	5	6
	Total	9	No animals	9	10	12
Mean SE severity	Non-telemetry			44.1 ± 5.1	47.5 ± 4.5	49.2 ± 2.8
	Telemetry			48.9 ± 2.4	50.3 ± 2.3	51.91 ± 1.4

### 2.4. Transmitter device implantation, video-electroencephalogram acquisition, and quantification of epileptiform spikes and spontaneous recurrent seizures

Initially, 34 animals were implanted with the CTA-F40 PhysioTel™ telemetry devices (Data Science International, Minneapolis, United States) before DFP administration as previously described (Puttachary et al., 2016; Putra et al., 2020). Hereafter, these animals are referred to as “telemetry animals.” Following recovery from surgery, the animals were housed individually and placed on PhysioTel receivers (RPC-1) connected to the Data Exchange Matrix 2.0 (DSI). Video-encephalography (vEEG) was recorded using Ponemah Acquisition software. NeuroScore 3.4.0 software was used to analyze EEG traces. A baseline EEG was recorded for a day and night (24 h) to normalize the post-DFP EEG for the detection of electrographic seizures and epileptiform spiking patterns. Individual threshold values for spike amplitude were set for each animal from the respective baseline EEG and then the number of spikes above the threshold were counted. Electrographic seizures were identified on the EEG traces using automated seizure detection feature in NeuroScore software with predetermined criteria, with above threshold spikes lasting at least 20s with minimum intervals of 0.05 s and maximum intervals of 1 s. All seizure events detected by the software were manually verified for behavioral convulsive seizure (CS) from the integrated video by an experimenter blinded to the treatment groups. Electrical noise, exploratory behavior, and grooming were identified and excluded from epileptiform spike analysis as previously described (Tse et al., 2014; Puttachary et al., 2016; Sharma et al., 2018).

### 2.5. *Status epilepticus* severity and seizure scoring after exposure to diisopropylfluorophosphate

The total duration (minutes) of convulsive seizures (≥ stage 3) during the 60 min between DFP and MDZ injections is defined as behavioral SE score. Behavioral SE was scored on a modified Racine scale as described previously (Racine, 1972; Puttachary et al., 2016; Putra et al., 2020). Animals following DFP injection were directly observed, and seizures scored by two experimenters. EEG with integrated video recording was also acquired and scored from animals implanted with telemetry devices in addition to behavioral SE scoring.

### 2.6. Immunohistochemistry, imaging and cell quantification

Animals were euthanized either on day 8 or 15 post DFP. Animals were perfused under terminal anesthesia with 4% paraformaldehyde and the brain was dissected. The blood was collected before perfusion. The brains were then kept in 4% paraformaldehyde for 24 h followed by 25% sucrose for another 72 h at 4°C. Perfused and cryoprotected brains were embedded in gelatin, flash frozen in liquid nitrogen cooled isopentane, and the tissue blocks were stored at −80°C. Brains were cut at 16 μM coronal sections using a cryostat (Thermo Fisher Scientific) and mounted over a glass slide. Each slide contains five brain sections each approximately 480 μM apart, representing rostral to caudal regions. Unstained and stained slides were stored at −20°C and 4°C, respectively. For IHC, the sections were subjected to antigen retrieval in citric acid buffer at 95°C for 23 min. Following antigen retrieval, slides were transferred to Shandon racks and washed with PBS (x5), blocked with 10% normal donkey serum for 1 h at room temperature before incubating with primary antibodies overnight at 4°C. Following a wash with PBS (x5) after primary antibody treatment, sections were incubated with fluorochrome-conjugated secondary antibodies for 1 h at room temperature, washed with PBS (x5), and mounted with a medium containing DAPI (VectaShield). The primary and secondary antibodies, their source, and dilution details are listed in Table 2. The Lecia DMi8 inverted fluorescence microscope equipped with Leica K5 passive cooled sCMOS camera was used to image the sections. Five brain sections from each animal were used to image different regions of the brain. Total number of immunopositive cells from each brain region per section were counted, averaged, and plotted graphs separately (per region) as counts. A linear mixed model analysis was also performed to investigate the overall effects in the brain due to exposure to DFP and mitigation by 1400W.

**Table 2:** Antibodies used for IHC.

**Primary antibody**	**Source**	**Catalogue number**	**Dilution factor**
Anti-IBA1 (goat)	Abcam	Ab5076	1:300
Anti-GFAP (mouse)	Sigma Aldrich	G3893	1:300
Anti-NeuN (rabbit)	EMD Millipore	ABN78	1:200
**Secondary antibody**	**Source**	**Catalogue number**	**Dilution factor**
AMCA blue streptavidin	Jackson ImmunoResearch	016–150-084	1:50
Biotinylated Donkey anti-Goat	Jackson ImmunoResearch	711–295-152	1:300
AlexaFluor 488 anti-mouse	Jackson ImmunoResearch	705–065-147	1:100
Rhodamine Red X anti-rabbit	Jackson ImmunoResearch	715–545-150	1:100

### 2.7. NeuN-FJB staining

To determine the extent of neurodegeneration, NeuN immunostained brain sections were further processed for FJB staining. The sections were first incubated in 0.006% potassium permanganate for 5 min with gentle shaking followed by three washes in distilled water for a minute each. The slides were incubated in 0.0003% FJB-0.1% acetic acid solution for 10 min in the dark followed by three washes in distilled water for a minute each. The slides were air-dried in the dark at RT, cleared in xylene and then mounted in Surgipath acrytol (Surgipath, Leica Biosystems, IL) and imaged. Colocalized neurons with NeuN (red) and FJB (green) were manually counted in five sections. Mean cell counts were plotted and quantified.

### 2.8. Cytokine detection in serum by ELISA

Blood was collected under terminal anesthesia and serum was separated and stored at −80°C. IL-1β, IL-6, TNF-α, and MCP-1 levels in serum were determined using RayBio® ELISA kits as per manufacturer’s instructions. All samples were run in duplicates along with positive and negative controls in a 96-well plate. A standard curve was plotted from lyophilized pure protein to determine sample concentrations. Change in color was analyzed by measuring absorbance at 450 nm using Spectramax M2 (Molecular devices) microplate reader. The values were expressed in picogram/mL, and data was analyzed by using GraphPad Prism software.

### 2.9. Griess assay for nitrite detection in serum

Griess assay is a colorimetric method of quantifying nitrite concentration in biological samples by using Griess reagents. In 50 μL of Griess reagent (Sigma-Aldrich®, St. Louis, MO, United States) was added to the serum samples in a 96-well plate (1:1) and incubated for 10 min until the development of color. Sodium nitrite was used to get a standard curve and finally absorbance was measured at 540 nm with Spectramax M2 (Molecular devices) microplate reader. The nitrite concentrations of the unknown samples were expressed in μM.

### 2.10. ROS detection in serum

ROS levels in the serum were detected by the OxiSelect™ Intracellular ROS Assay Kit and manufacturer’s instructions were followed. The fluorescence intensity was measured using excitation/emission wavelength at 480/530 nm, respectively, with Spectramax M2 (Molecular devices) microplate reader. The fluorescence value from the control groups was subtracted as background and the results were expressed as relative fluorescence values (RFUs).

### 2.11. Glutathione assay

GSH and GSSG (oxidized GSH) serum levels were analyzed by using the Glutathione Colorimetric Detection Kit (Thermo Fisher Scientific, Carlsbad, CA, United States) as per manufacturer’s instructions. All samples were run in duplicates along with positive and negative controls in a 96-well plate. 2-vinylpyridine (2-VP) was used to block color development by un-oxidized glutathione (GSH) and thus measured the concentration of only oxidized glutathione (GSSG). Further, total glutathione (GSH + GSSG) was measured when no blocking was done with 2-VP. With this method, free GSH (un-oxidized) was calculated by subtracting the GSSG values from the total glutathione (GSH + GSSG) values. The final concentrations were read at 405 nm using a Spectramax M2 (Molecular devices) microplate reader and expressed in μM. Concentrations for free GSH, GSSG, and ratio of GSH to GSSG were plotted and analyzed.

### 2.12. Experimental design, rigor and statistical analysis

All experiments were adhered to rigorous blinding and group randomization. We chose statistical tests during the design phase and used GraphPad Prism v8 for routine. The Shapiro–Wilk test was used to determine the normality of each dataset. No data points were excluded. When comparing multiple groups, the significance of the data was detected using one-way ANOVA with Tukey’s post-hoc test. Data within two factors were analyzed either with two-way ANOVA or repeated measure two-way ANOVA with Tukey’s multiple comparisons. For descriptive statistical analysis please refer to Table 3.

**Table 3:** Statistical tests with mean ± SEM values.

**Figure 2**	**DFP + Veh**	**1400W (10 mg/kg)**	**1400W (15 mg/kg)**	**Statistical analysis**		
A	3.778 ± 0.8098	8.771 ± 2.321	6.7 ± 2.353	One-way ANOVA (Tukey’s post-hoc)		
	**Males**	**Females**				
C	8.47 ± 1.943	4.769 ± 1.428		Mann–Whitney test		
	**DFP + Veh**	**1400W (10 mg/kg)**	**1400W (15 mg/kg)**	**Statistical analysis**		
E	3.821 ± 1.428	2.955 ± 0.8982	3.104 ± 0.7513	One-way ANOVA (Tukey’s post-hoc)		
	**Males**	**Females**				
G	3.853 ± 0.9176	2.746 ± 0.7055		Mann–Whitney test		
**Figure** **3****C**	**Veh**	**Veh + 1400W**	**DFP + Veh**	**1400W (10 mg/kg)**	**1400W (15 mg/kg)**	**Statistical analysis**
AMY	117.6 ± 8.5	101.3 ± 7.945	147.4 ± 12.85	79.33 ± 7.064	85.98 ± 12.73	One-way ANOVA (Tukey’s post-hoc)
CA1	91.8 ± 7.08	71.12 ± 6.851	121.5 ± 15.15	57.11 ± 5.367	74.8 ± 8.806	
CA3	73.96 ± 4.915	57.39 ± 6.873	114.2 ± 14.52	57.74 ± 3.713	68.82 ± 6.177	Same as above
DG	88.40 ± 9.296	63 ± 8.293	122.8 ± 23.4	49.91 ± 3.12	70.81 ± 10.84	Same as above
PC	77.17 ± 3.858	76.94 ± 5.189	122.2 ± 11.35	91.97 ± 3.567	82.21 ± 7.06	Same as above
SUB	82.42 ± 11.56	72.38 ± 5.976	121.6 ± 15.14	50.37 ± 5.53	72.13 ± 9.28	Same as above
**Figure** **3****E**	**Veh**	**Veh + 1400W**	**DFP + Veh**	**1400W (10 mg/kg)**	**1400W (15 mg/kg)**	**Statistical analysis**
AMY	63.63 ± 6.537	64.38 ± 10.39	118.8 ± 16.06	81.07 ± 4.315	94.44 ± 13.4	One-way ANOVA (Tukey’s post-hoc)
CA1	37.53 ± 2.931	37.52 ± 6.862	95.42 ± 14.47	78.89 ± 8.86	56.47 ± 7.757	
CA3	35.15 ± 3.073	30.33 ± 6.872	91.36 ± 11.21	73.52 ± 8.53	58.67 ± 9.144	Same as above
DG	39.77 ± 6.933	40.96 ± 6.206	99.96 ± 15.86	81.69 ± 17.31	76.12 ± 11.55	Same as above
PC	49.44 ± 4.298	47.5 ± 2.842	91.16 ± 13.37	79.5 ± 9.122	88.67 ± 12.18	Same as above
SUB	39.54 ± 2.652	40.38 ± 6.235	105.2 ± 15.72	98.33 ± 14.99	66.92 ± 7.384	Same as above
**Figure** **4****C**	**Veh**	**DFP + Veh**	**1400W (10 mg/kg)**	**1400W (15 mg/kg)**	**Statistical analysis**	
AMY	35.78 ± 8.66	96.22 ± 7.394	61.73 ± 7.923	51.39 ± 5.776	One-way ANOVA (Tukey’s post-hoc)	
CA1	10.22 ± 2.936	85.58 ± 15.77	52.15 ± 8.517	49.63 ± 5.002		
CA3	9.593 ± 3.064	83.69 ± 17.69	31.58 ± 4.879	43.46 ± 6.755	Same as above	
DG	14.89 ± 2.174	74.75 ± 13.39	43.52 ± 6.89	40.67 ± 4.425	Same as above	
PC	24.19 ± 4.926	86.11 ± 9.363	55.3 ± 7.061	51.37 ± 8.085	Same as above	
SUB	13.19 ± 2.244	99.25 ± 14.4	62.05 ± 11.08	64.22 ± 5.718	Same as above	
**Figure** **4****E**	**Veh**	**DFP + Veh**	**1400W (10 mg/kg)**	**1400W (15 mg/kg)**	**Statistical analysis**	
AMY	17.74 ± 1.921	97.13 ± 9.21	62.83 ± 6.375	55.14 ± 4.736	One-way ANOVA (Tukey’s post-hoc)	
CA1	11.78 ± 1.607	77.08 ± 13.8	52.25 ± 7.042	49.03 ± 3.903		
CA3	14.78 ± 2.29	72.9 ± 6.24	50.58 ± 6.97	46.9 ± 4.31	Same as above	
DG	13.8 ± 1.53	60.9 ± 4.48	43.9 ± 6.54	37.8 ± 3.93	Same as above	
PC	7.67 ± 2.06	81.2 ± 5.84	73.6 ± 4.06	72.76 ± 6.26	Same as above	
SUB	13.19 ± 2.2	99.25 ± 14.4	62.05 ± 11.08	64.22 ± 5.72	Same as above	
**Figure** **5****C**	**Veh**	**Veh + 1400W**	**DFP + Veh**	**1400W (10 mg/kg)**	**1400W (15 mg/kg)**	**Statistical analysis**
AMY	12.74 ± 5.559	11.88 ± 5.427	66.98 ± 13.41	70.19 ± 10.17	41.92 ± 6.726	2-way ANOVA (Tukey’s post-hoc)
CA1	1.952 ± 0.6792	2.688 ± 0.834	22.02 ± 4.607	18.43 ± 5.96	12.78 ± 4.281	
CA3	1.929 ± 1.302	3.583 ± 1.711	20.95 ± 3.49	16.92 ± 3.849	11.18 ± 4.148	Same as above
DG	8.869 ± 2.309	2.667 ± 1.483	30.44 ± 4.546	20.85 ± 6.123	13.02 ± 3.004	Same as above
PC	14.4 ± 8.511	4.729 ± 2.412	63.68 ± 13.42	58.78 ± 5.402	29.4 ± 4.36	Same as above
SUB	5.107 ± 2.789	3.813 ± 1.323	32.23 ± 8.333	39.97 ± 7.08	9.931 ± 3.173	Same as above
**Figure** **6****C**	**Veh**	**DFP + Veh**	**1400W (10 mg/kg)**	**1400W (15 mg/kg)**	**Statistical analysis**	
AMY	3.9 ± 1.54	29.8 ± 5.97	16.84 ± 3.32	17.85 ± 3.34	One-way ANOVA (Tukey’s post-hoc)	
CA1	1.11 ± 0.38	18.6 ± 3.53	13.51 ± 3.73	10.6 ± 1.46		
CA3	0.865 ± 0.6	23.6 ± 2.51	22.9 ± 5.82	16.26 ± 1.89	Same as above	
DG	4.56 ± 1.17	28.36 ± 3.02	18.3 ± 3.72	18.75 ± 2.43	Same as above	
PC	6.53 ± 3.96	21.17 ± 2.7	21.64 ± 4.84	16.5 ± 2.21	Same as above	
SUB	3.66 ± 2.51	18.8 ± 3.18	15.45 ± 2.95	11.1 ± 1.88	Same as above	
**Figure** **7**	**Veh**	**DFP + Veh**	**1400W (10 mg/kg)**	**1400W (15 mg/kg)**	**Statistical analysis**	
A (IL-1β)	17.94 ± 1.897	496.7 ± 9.707	252.5 ± 35.63	36.21 ± 15.76	One-way ANOVA (Tukey’s post-hoc)	
A (IL-6)	177.9 ± 34.77	2,408 ± 101.9	2,211 ± 203	1745 ± 413	Same as above	
A (TNF-α)	28.98 ± 6.934	128.1 ± 1.938	47.04 ± 11.74	32.06 ± 6.708	Same as above	
A (MCP-1)	756.1 ± 227.1	2092 ± 164.3	1857 ± 357.9	1,163 ± 114.7	Same as above	
B (IL-1β)	17.94 ± 1.897	373.5 ± 59.14	144.3 ± 23.2	31.95 ± 3.336	Same as above	
B (IL-6)	177.9 ± 34.77	3,117 ± 80.48	2090 ± 102.3	1,284 ± 236.4	Same as above	
B (TNF-α)	28.98 ± 6.934	90.16 ± 12.95	40.38 ± 9.899	20.10 ± 6.538	Same as above	
B (MCP-1)	756.1 ± 227.1	2,368 ± 310.8	798.6 ± 121.7	756.1 ± 227.1	Same as above	
**Figure** **8**	**Veh**	**DFP + Veh**	**1400W (10 mg/kg)**	**1400W (15 mg/kg)**	**Statistical analysis**	
A (GSH)	22.68 ± 0.05766	12.37 ± 1.179	12.72 ± 0.08997	11.84 ± 0.08225	Same as above	
A (GSSG)	2.377 ± 0.03279	6.371 ± 0.560	5.48 ± 0.4314	5.702 ± 0.6281	Same as above	
A (ratio)	9.557 ± 0.156	1.993 ± 0.2131	2.472 ± 0.2748	2.385 ± 0.4249	Same as above	
B (GSH)	22.68 ± 0.05766	13.17 ± 1.23	13.11 ± 0.4957	14.73 ± 0.4545	Same as above	
B (GSSG)	2.377 ± 0.03279	7.057 ± 0.4937	4.938 ± 0.5015	3.645 ± 0.2794	Same as above	
B (ratio)	9.557 ± 0.156	1.896 ± 0.1713	2.902 ± 0.3714	4.297 ± 0.4809	Same as above	
**Figure** **9**	**Veh**	**DFP + Veh**	**1400W (10 mg/kg)**	**1400W (15 mg/kg)**	**Statistical analysis**	
A	16.54 ± 3.856	56.85 ± 6.144	52.23 ± 5.715	19.64 ± 5.116	Same as above	
B	181.3 ± 22.43	304.7 ± 44.15	No data	157.4 ± 20.83	Same as above	
C	16.54 ± 3.856	59.79 ± 6.785	55.06 ± 6.096	17.95 ± 2.319	Same as above	

## 3. Results

### 3.1. *Status epilepticus* severity comparison between vehicle and 1400W treated groups (10 and 15 mg/kg)

**Figure 2:**
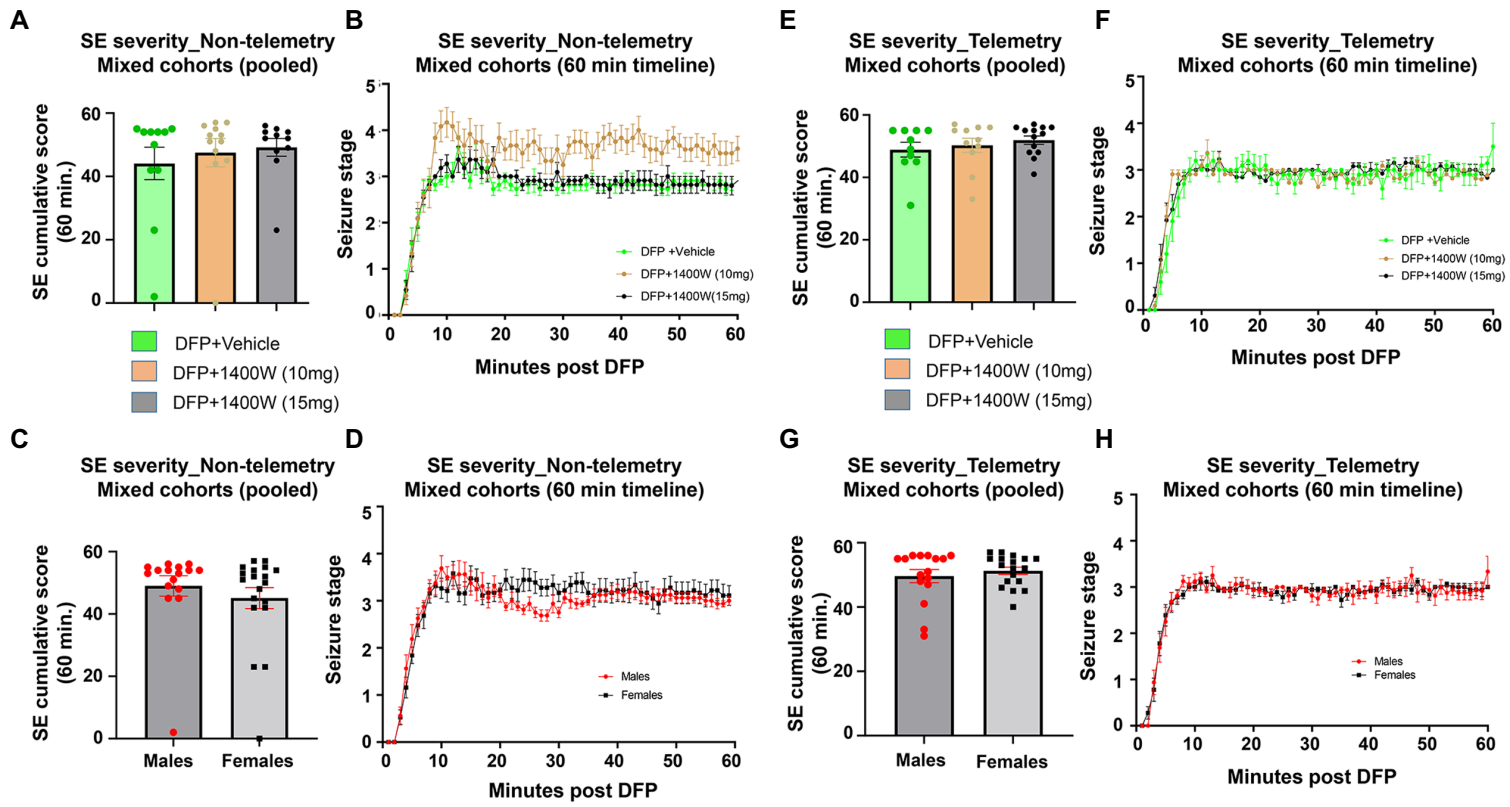
SE severity quantification during the 60 min post DFP in non-telemetry and telemetry groups. There were no significant differences in the SE severity between the vehicle and 1400W treated groups in both sexes. One-way ANOVA with Tukey’s post-hoc for multiple comparisons (**A,B**; *n* = 11–12), (**C,D**; *n* = 16–19), (**E,F**; *n* = 10–13), and (**G,H**; *n* = 16–18).

There were no significant differences in the initial SE severity between the vehicle and 1400W treated groups in either telemetry or non-telemetry experimental groups. Initial SE severity of all males and females that were later treated with vehicle or 1400W were also compared and no significant differences in SE severity were observed (Figure 2). Further, there were also no sex differences in SE severity within groups.

### 3.2. 1400W reduced diisopropylfluorophosphate induced gliosis at 8-day and 15-day post diisopropylfluorophosphate

**Figure 3:**
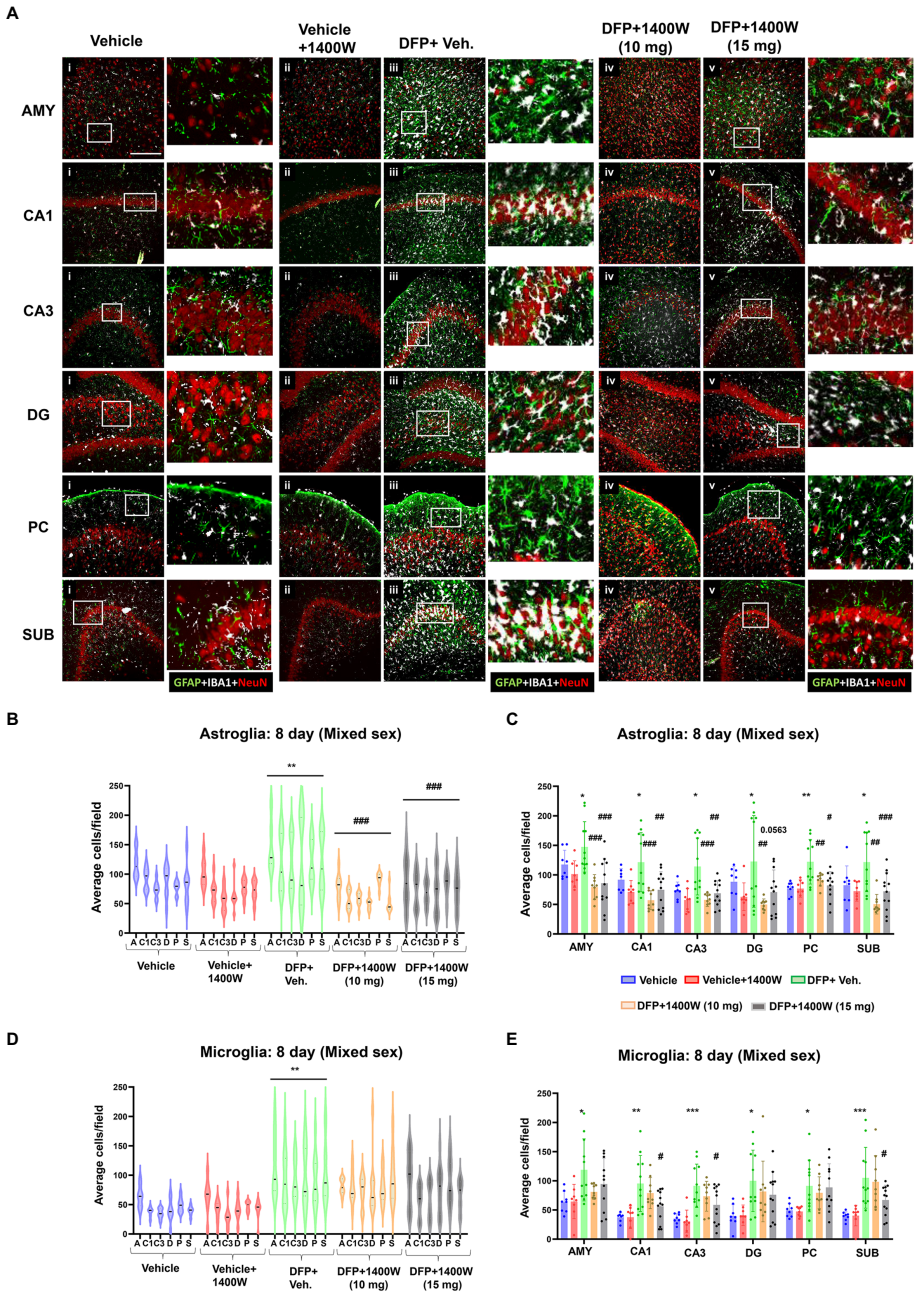
The effects of 1400W treatment for a week on DFP-induced astrogliosis and microgliosis. Representative images of GFAP (green, for astrocytes), IBA1 (gray, for microglia), and NeuN (red, for neurons) positive cells representing Amygdala (AMY), CA1, CA3, Dentate gyrus (DG), Piriform Cortex (PC), Subiculum (SUB) regions of the brains from control, vehicle+1400W, DFP + vehicle, DFP + 1400W (10 and 15 mg/kg) groups **(A)**. The representative enlarged images for each group and the brain regions are shown in the adjacent columns, scale bar = 50 μM. The mixed effects model revealed a significant increase in GFAP+ve cells in brain as a whole in DFP + vehicle group and 1400W at both 10 and 15 mg/kg significantly reduced the GFAP positive cells **(B)**. Two-way ANOVA showed an increase in GFAP positive cells within different regions of the brain following DFP exposure and 1400W reduced the DFP-induced astrogliosis **(C)**. The mixed effects model revealed a significant increase in microgliosis in DFP + vehicle group but 1400W at either of the concentration had no significant effect. However, two-way ANOVA analysis of individual brain regions showed a significant reduction of microgliosis at 15 mg/kg in CA1, CA3 and SUB regions **(D,E)**, *n* = 8–12. Repeated measure Two-way ANOVA (mixed effects) with Tukey’s post-hoc for multiple comparisons. * Represents DFP effect compared to vehicle (control); # represents the 1400W effect compared to DFP + vehicle group, * represents DFP effect compared to the vehicle (control) and # represents 1400W effect compared to DFP + vehicle group. *p < 0.05, **p < 0.01, ***p < 0.001, #*p* < 0.05, ##*p* < 0.01, ###*p* < 0.001.

**Figure 4:**
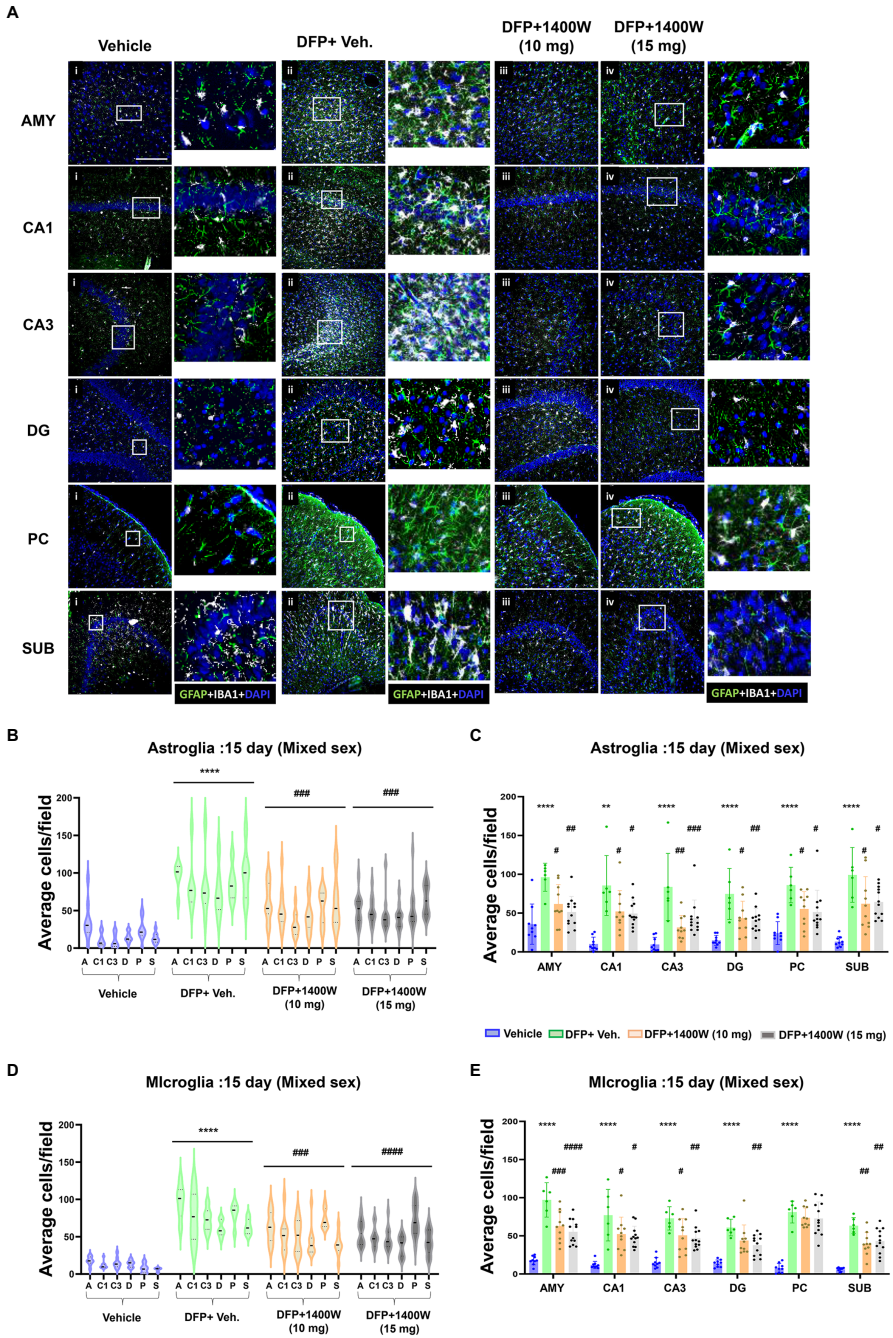
The effects of 1400W treatment for 2 weeks on DFP-induced astrogliosis and microgliosis. Representative images of GFAP (green, for astrocytes) and IBA1 (gray, for microglia) positive cells representing Amygdala (AMY), CA1, CA3, Dentate gyrus (DG), Piriform Cortex (PC), Subiculum (SUB) regions of the brains from control, vehicle+1400W, DFP + vehicle, DFP + 1400W (10 and 15 mg/kg) groups **(A)**. The representative enlarged images for each group and the brain regions are shown in the adjacent columns, scale bar = 50 μM. The mixed effects model revealed a significant increase in both GFAP+ve and IBA1 + ve cells in brain as a whole in the DFP + vehicle group. 1400W at both 10 and 15 mg/kg significantly reduced astrogliosis and microgliosis **(B,D)**. Two-way ANOVA showed an increase in GFAP positive cells within different regions of the brain following DFP exposure. 1400W at both concentrations reduced the DFP-induced astrogliosis in all brain regions. However, at 10 mg microgliosis was not reduced in the DG and PC **(C,E)**, *n* = 6–12. Repeated measure Two-way ANOVA (mixed effects) with Tukey’s post-hoc for multiple comparisons. * Represents DFP effect compared to the vehicle (control); # represents the 1400W effect compared to DFP + vehicle group * represents DFP effect compared to vehicle (control) and # represents 1400W effect compared to DFP + vehicle group. *p < 0.05, **p < 0.01, ***p < 0.001, #*p* < 0.05, ##*p* < 0.01, ###*p* < 0.001.

Brain section from all groups were stained simultaneously with anti IBA1 (microglial marker) and anti GFAP (astroglial marker). Representative images from the amygdala (AMY), CA1, CA3, dentate gyrus (DG), piriform cortex (PC) and subiculum (SUB) are shown (Figures 3, 4). IBA1 and GFAP positive cell counts were plotted for 8-day (Figures 3B–E) and 15-day (Figures 4B–E) post DFP. Average cell counts were compared between groups as mixed sex cohorts. Repeated measure analysis in a mixed effects model revealed that DFP + vehicle group as a whole with all brain regions had significantly a greater number of astroglia and microglia when compared to control groups at 8-day (Figures 3B,D) and 15-day (Figures 4B,D) post DFP. 1400W treatment at both 10 and 15 mg/kg for either one week or two weeks significantly reduced the number of astroglia (Figures 3B, 4B). Interestingly, short-term treatment with 1400W for a week at both 10 mg/kg and 15 mg/kg did not significantly reduce the DFP-induced microgliosis (Figure 3D). In contrast, two weeks treatment significantly reduced the DFP-induced microgliosis (Figure 4D). Two-way ANOVA analysis also showed a similar trend when the brain regions were independently analyzed for astrogliosis (Figures 3C, 4C) and microgliosis (Figures 3E, 4E). In summary, 1400W treatment for two weeks at 15 mg/kg was effective in reducing the DFP-induced astrogliosis and microgliosis. No sex differences were observed in either DFP-induced astrogliosis and microgliosis or the mitigating effects by 1400W in both treatment regimens (Supplementary Figures S1, S2).

### 3.3. 1400W reduces diisopropylfluorophosphate induced neurodegeneration at 8-day and 15-day post diisopropylfluorophosphate

**Figure 5:**
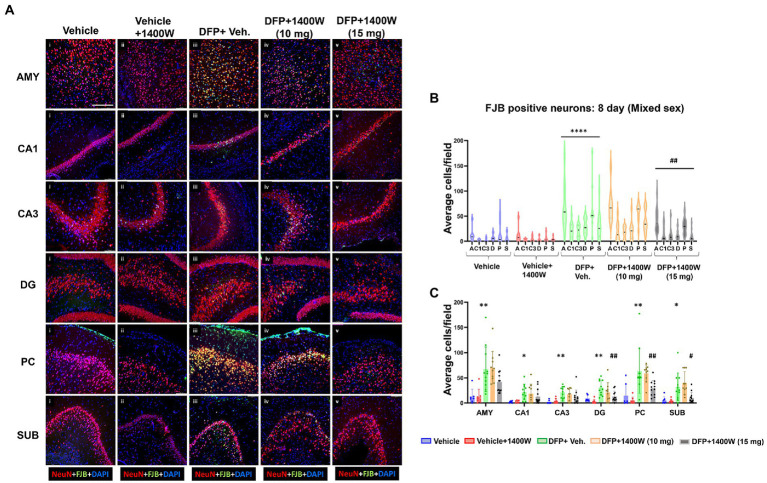
The effects of 1400W treatment for a week on DFP-induced neurodegeneration. Representative images of NeuN (red, neurons) and FJB (green) positive cells from Amygdala (AMY), CA1, CA3, Dentate gyrus (DG), Piriform Cortex (PC), Subiculum (SUB) regions are shown. Co-localized (orange) cells represent degenerating neurons. Sections were counterstained with DAPI (blue) to represent all nuclei, scale bar = 50 μM **(A)**. Mixed effects model revealed a significant increase in degenerating neurons in brain as a whole in DFP + vehicle group and 1400W at 15 mg/kg significantly reduced the FJB positive neurons **(B)**.The two-way ANOVA analysis showed an increase in FJB positive neurons within different regions of the brain following DFP treatment and 1400W treatment significantly reduced FJB positive neurons at 15 mg/kg in DG, PC and SUB **(C)**. *n* = 7–12, Repeated measure Two-way ANOVA (mixed effects) with Tukey’s post-hoc for multiple comparisons. * Represents DFP effect compared to the vehicle (control); # represents the 1400W effect compared to DFP + vehicle group * represents DFP effect compared to vehicle (control) and # represents 1400W effect compared to DFP + vehicle group. *p < 0.05, **p < 0.01, ***p < 0.001, #*p* < 0.05, ##*p* < 0.01, ###*p* < 0.001.

**Figure 6:**
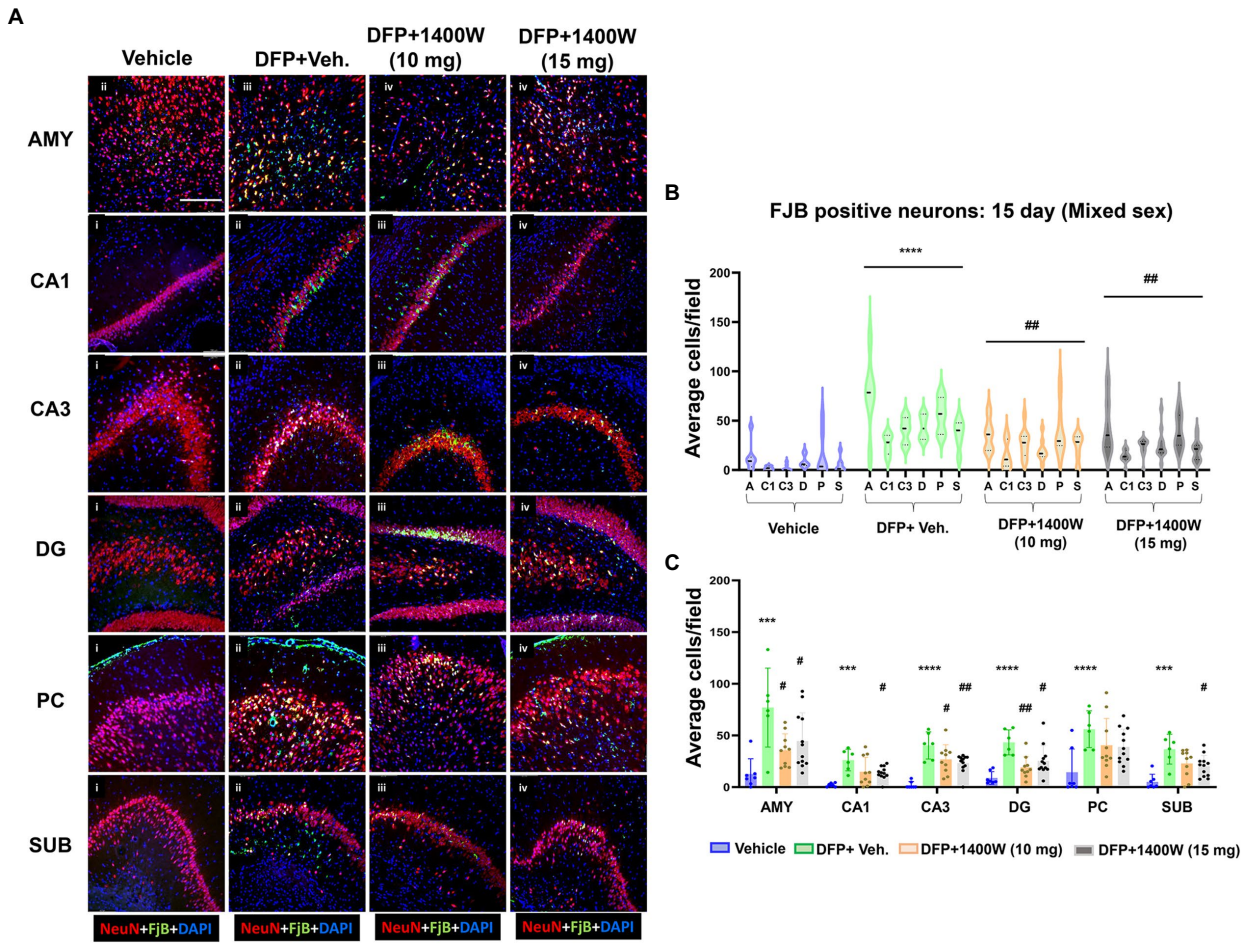
The effects of 1400W treatment for 2 weeks on neurodegeneration. Representative images of NeuN (red, for neurons) and FJB (green) positive cells from Amygdala (AMY), CA1, CA3, Dentate gyrus (DG), Piriform Cortex (PC), Subiculum (SUB) regions are shown. Co-localized (orange) cells represent degenerating neurons. Sections were counterstained with DAPI (blue) to represent all nuclei, scale bar = 50 μM **(A)**. Mixed effects model revealed a significant increase in degenerating neurons in brain as a whole in DFP + vehicle group at 15 days post-exposure **(B)**.The two-way ANOVA analysis showed an increase in FJB positive neurons within different regions of the brain following DFP exposure and 1400W treatment significantly reduced the DFP induced increase in FJB positive neurons at 10 mg/kg in AMY, CA3 and DG, while 15 mg/kg reduced in all regions except the PC **(C)**, *n* = 7–12. Repeated measure Two-way ANOVA (mixed effects) with Tukey’s post-hoc for multiple comparisons. * Represents DFP effect compared to vehicle (control); # represents the 1400W effect compared to DFP + vehicle group * represents DFP effect compared to vehicle (control) and # represents 1400W effect compared to DFP + vehicle group. *p < 0.05, **p < 0.01, ***p < 0.001, #*p* < 0.05, ##*p* < 0.01, ###*p* < 0.001.

Brain section from all groups were first stained with anti NeuN (neuronal marker) and then with FJB stain (marker for degenerating cells). The sections were also counterstained with DAPI to mark all nuclei. Representative images from the AMY, CA1, CA3, DG, PC and SUB are shown for 8-day (Figure 5A) and 15-day (Figure 6A) post DFP. Repeated measure analysis in a mixed effects model revealed that DFP + vehicle group as a whole with all brain regions had significantly a greater number of degenerating neurons (FJB + NeuN colocalized cells) when compared to control groups at both 8-day (Figure 5B) and 15-day (Figure 6B) post DFP. 1400W at 10 mg/kg was effective in reducing neurodegeneration when it was administered daily for 2 weeks but not for a week (Figures 5B, 6B). In contrast, 15 mg/kg significantly reduced the number of degenerating neurons at both time points when it was administered for a week or 2 weeks (Figures 5B, 6B). Two-way ANOVA analysis for both concentrations and duration for each brain region was also analyzed and found a similar trend as in the mixed effects model (Figures 5C, 6C). No significant sex differences were observed in neurodegeneration (Supplementary Figures S1, S2).

### 3.4. 1400W reduces diisopropylfluorophosphate-induced proinflammatory cytokines in the serum

**Figure 7:**
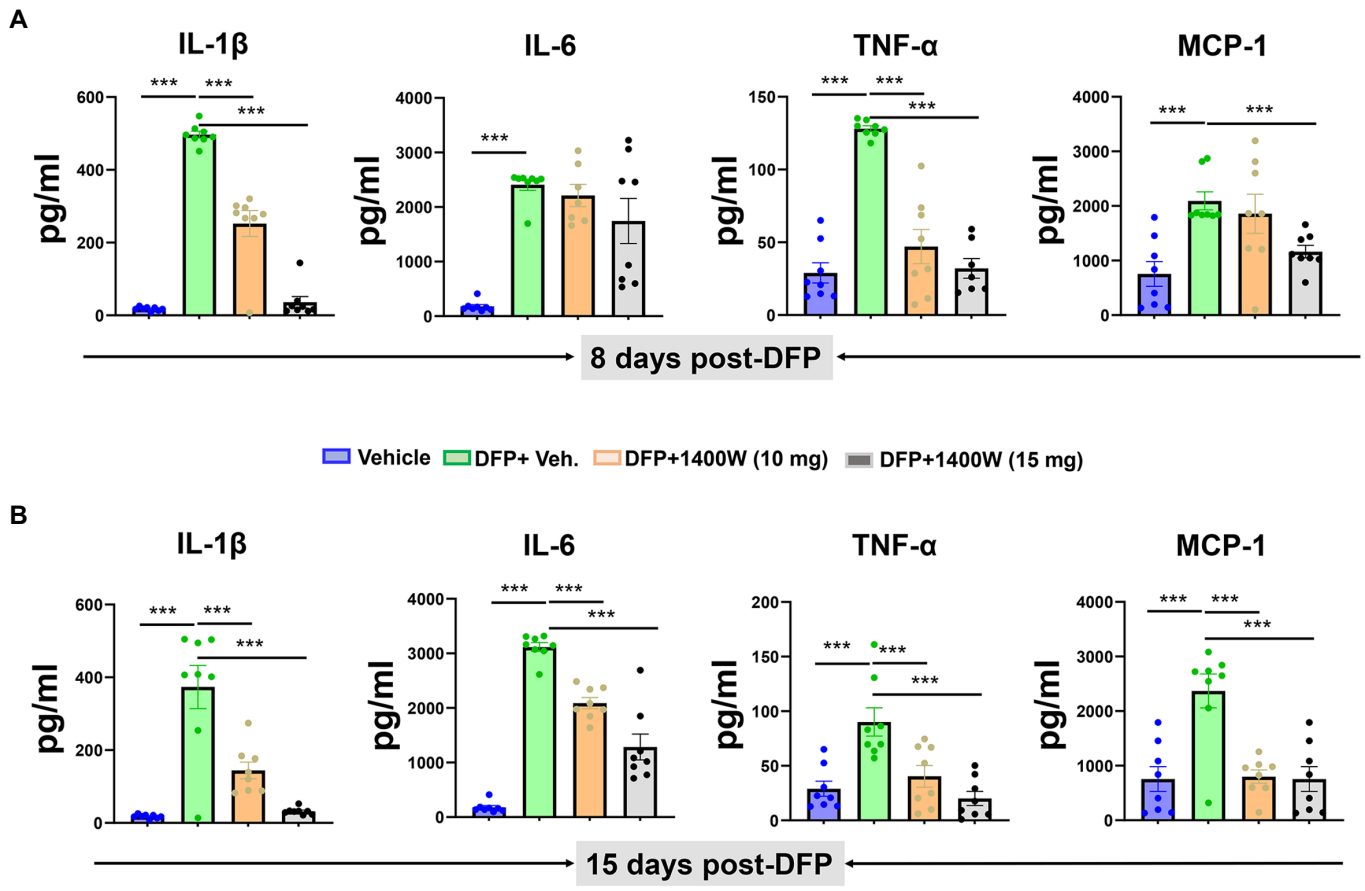
The effects of 1400W on the DFP-induced serum pro-inflammatory cytokines. At 8-day post DFP, 1400 W at 10 mg/kg significantly reduced the DFP induced IL-1β and TNF-α but not MCP-1, while 1400W at 15 mg/kg significantly reduced DFP induced IL-1β, IL-6, TNF-α and MCP-1 **(A)**. At 15-day post DFP, 1400 W both at (10 mg/kg and 15 mg/kg) significantly reduced the upregulated cytokine levels following DFP exposure **(B)**, *n* = 8, one-way ANOVA with Tukey’s post-hoc for multiple comparisons; *p < 0.05, **p < 0.01, ***p < 0.001.

Cytokines in the serum were analyzed using a commercial ELISA kit. Pro-inflammatory cytokines such as IL-1β, IL-6, TNF-α and MCP-1 were probed at 8-day (Figure 7A) and 15-day (Figure 7B) post DFP. At both time-points post-DFP, IL-1β, IL-6, TNF-α and MCP-1 were significantly increased in the serum. 1400W treatment at 15 mg/kg for 1 or 2 weeks significantly reduced all cytokines (Figures 7A–C). In contrast, 10 mg/kg 1400W for a week had no significant effects on IL-6 and MCP-1 but 2 weeks of treatments reduced all cytokines (Figures 7A,B).

### 3.5. 1400W prevents diisopropylfluorophosphate-induced oxidation of GSH to GSSG in the serum

**Figure 8:**
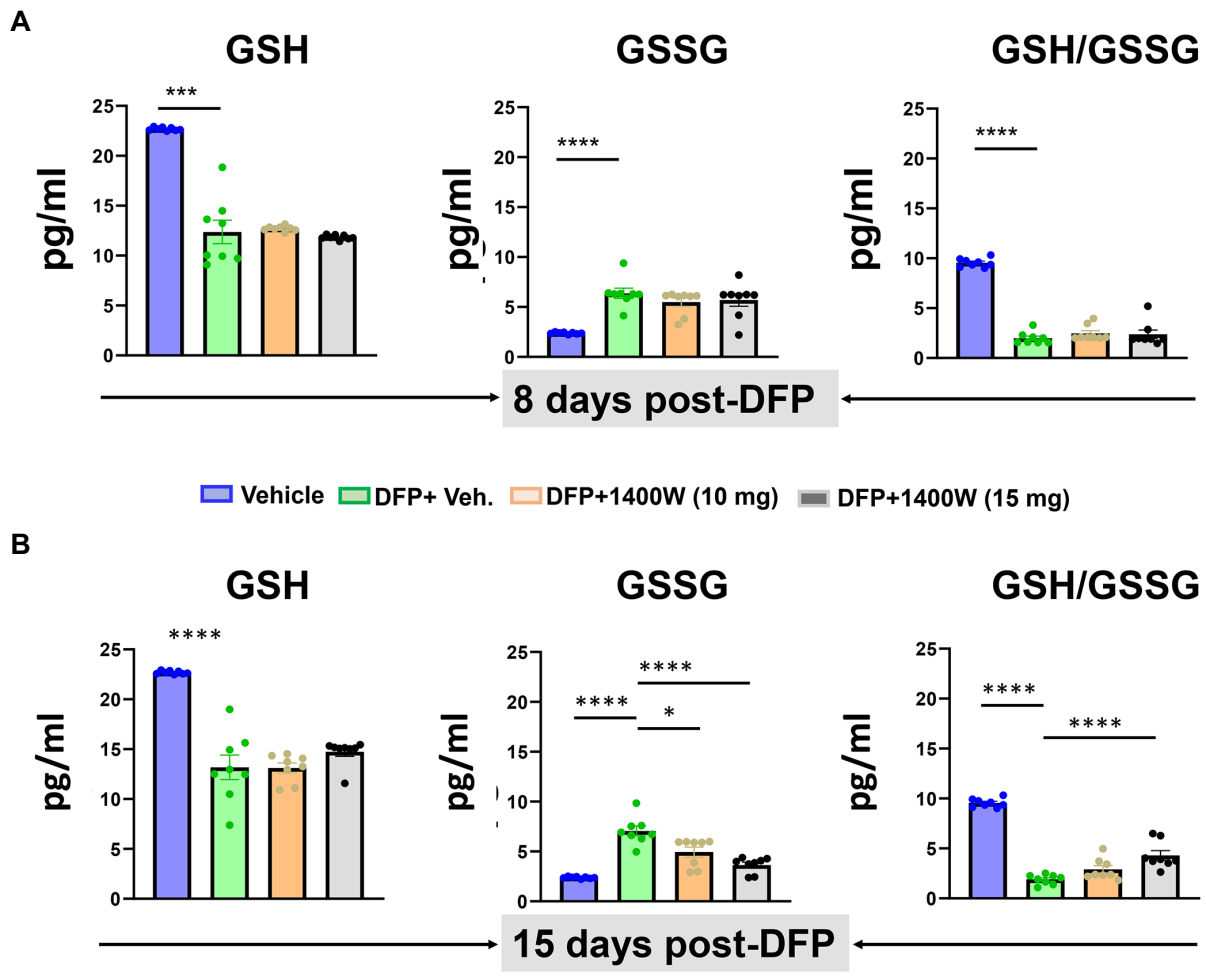
The effects of DFP and 1400W on serum glutathione profiles. At 8-day, 1400W (10 mg/kg or 15 mg/kg) had no significant effect on serum GSH and GSSG levels **(A)**. At 15-day post DFP, 1400 W (15 mg/kg) significantly reduced GSSG levels and also increased the GSH/GSSG ratio in DFP exposed rats **(B)**, *n* = 8, one-way ANOVA with Tukey’s post-hoc for multiple comparisons; *p < 0.05, **p < 0.01, ***p < 0.001.

Compared to controls at 8-day, DFP exposed animals showed significantly lower levels of GSH and higher levels of GSSG and thus an overall lower GSH:GSSG ratio was observed. 1400W treatment at 10 or 15 mg/kg for a week did not alter DFP-induced GSH and GSSG changes (Figure 8A). However, treating with 1400W at 15 mg/kg for 2 weeks significantly altered the DFP-induced effects (Figure 8B).

### 3.6. 1400W reduces diisopropylfluorophosphate-induced nitro-oxidative and oxidative stress in the serum following diisopropylfluorophosphate exposure

**Figure 9:**
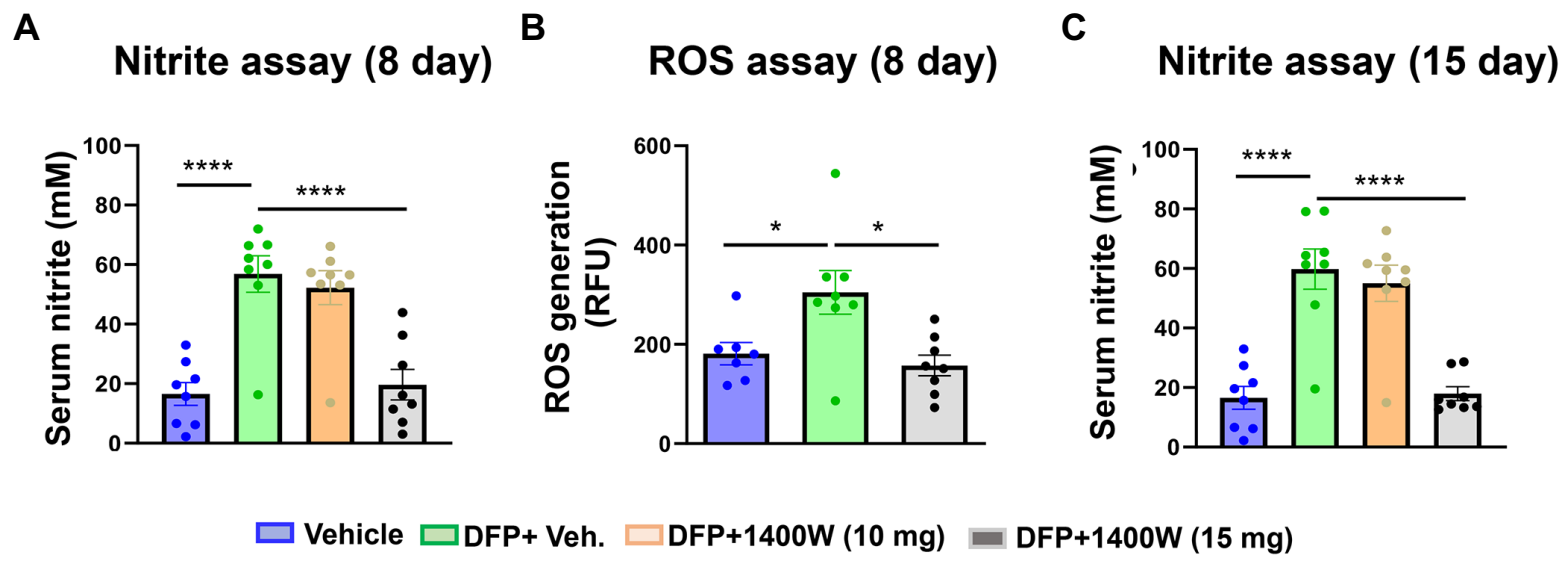
The effects of DFP and 1400W on serum nitro-oxidative stress markers. At 8-day post DFP, 1400 W (15 mg/kg) significantly reduced serum levels of nitrite and ROS levels in DFP exposed rats **(A,B)**. At 15-day post DFP, 1400 W (15 mg/kg) significantly reduced serum nitrite in DFP exposed rats **(C)**, *n* = 8, one-way ANOVA with Tukey’s post-hoc for multiple comparisons; *p < 0.05, **p < 0.01, ***p < 0.001.

Serum nitrite and ROS levels were analyzed using commercial ELISA kits. Serum levels of nitrite (Figure 9A) and ROS (Figure 9B) were found to be significantly elevated in animals exposed to DFP at both time points (Figure 9). 1400W at 15 mg/kg for a week or 2 weeks significantly reduced the nitrite and ROS levels (Figures 9A,B). Lower concentration of 10 mg/kg 1400W for a week or 2 weeks was ineffective.

### 3.7. 1400W had no effect on diisopropylfluorophosphate-induced epileptiform spikes and SRS

**Figure 10:**
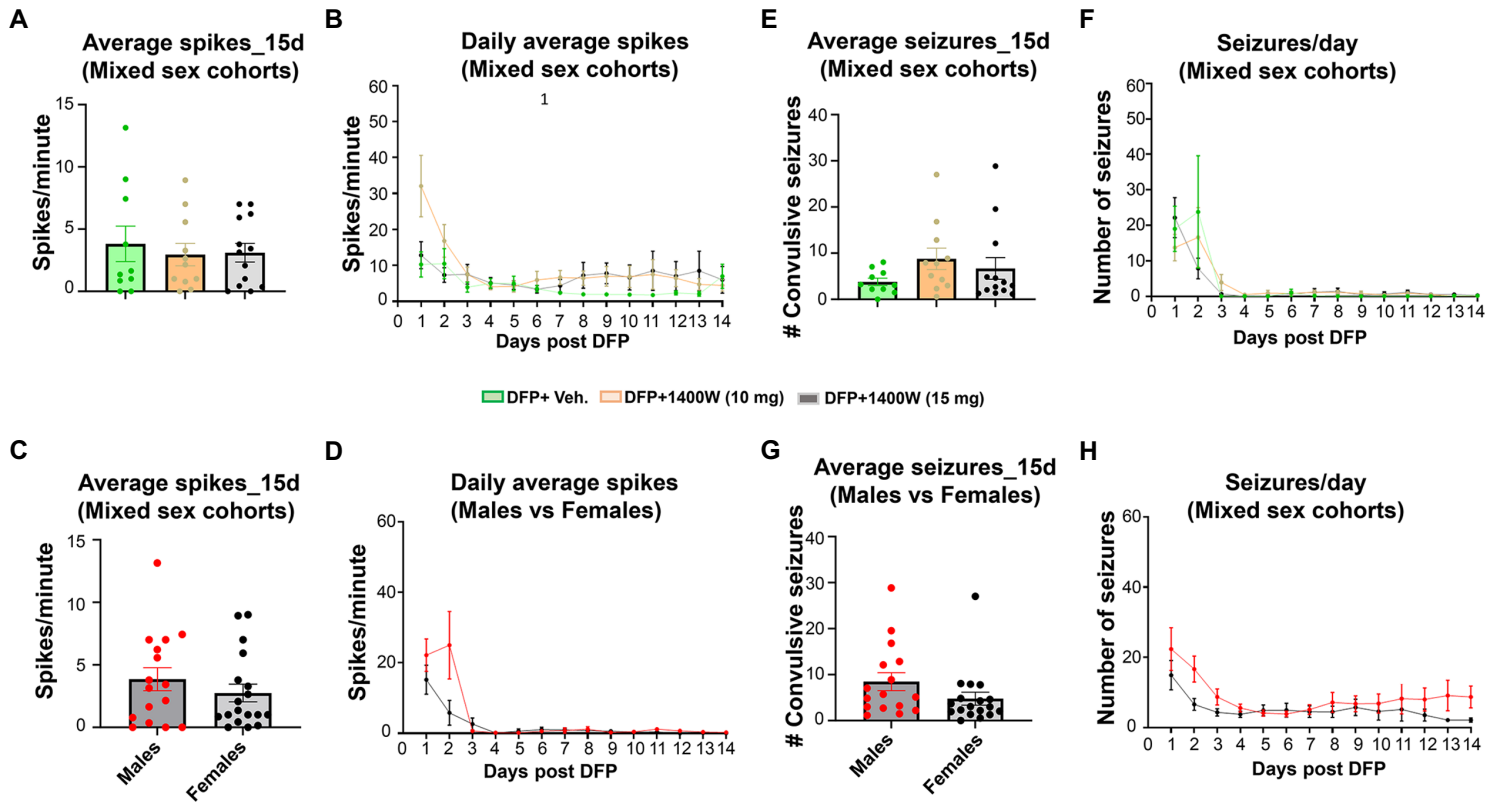
The effects of DFP and 1400W on epileptiform spikes and SRS during the 15 days post-exposure. Epileptiform spikes were compared in mixed sex cohorts and between males and females **(A–D)**. There were no significant differences in the epileptiform spike rate between any treatment groups in mixed sex cohorts **(A,B)** or between males and females **(C,D)**. Electrographic seizures were also compared in mixed sex cohorts and between males and females **(E–H)**. There were also no significant differences in the electrographic seizures (SRS) between any treatment groups in mixed sex cohorts **(E,F)** or between males and females **(G,H)**. One-way ANOVA with Tukey’s post-hoc for multiple comparisons. (**A,B**; *n* = 10–13), (**C,D**; *n* = 16–18), (**E,F**, *n* = 10–13), (**G,H**; *n* = 16–18).

Video-EEG was continuously monitored from the rats that were given vehicle or 1400W following DFP exposure. An SRS detected on EEG was manually verified with synchronized video. There were no significant differences in the epileptiform spike rate (Figures 10A–D) or RS frequency (Figures 10E–H) between any treatment groups in mixed sex cohorts or between males and females across all groups. There were no sex differences in SRS and epileptiform spike rate when they were independently analyzed within each treatment group (Supplementary Figure S3).

## 4. Discussion

The goal of this study was to optimize the efficacy of 1400W in all spectra of SE severity in the rat DFP model. We have previously demonstrated the long-term disease modifying effects of 1400W at higher concentration but for a shorter duration (20 mg/kg twice daily for the first 3 days post exposure) in the DFP induced neurotoxicity rat model with mild to moderate SE severity (Putra et al., 2020). A similar dosing regimen in the rat kainate model also mitigated SE-induced long-term brain injury (Puttachary et al., 2016). The protective effects of 1400W in other neurodegenerative disorders including traumatic brain injury models have also been demonstrated (Jafarian-Tehrani et al., 2005; Ryu and McLarnon, 2006; Kumar et al., 2014). In this study, we investigated the short-term effects of two lower concentrations of 1400W (10 and 15 mg/kg) when administered daily for a week or 2 weeks after acute exposure to DFP. OP intoxication is a public health concern and also pose a great risk to military personnel and civilian life in war prone areas (Jeyaratnam, 1990; Yanagisawa et al., 2006; Kloske and Witkiewicz, 2019). Current drug therapies are symptomatic and only reduce seizures and mortality. In addition to medical counter measures (MCMs) such as atropine, oximes and benzodiazepine therapy, general supportive measures like exogenous oxygen supply and intravenous fluids have been used in primary care for more than 60 years (Eddleston et al., 2008; Clossen and Reddy, 2017). Though the MCMs reduce the lethal effects of OPNA poisoning they do not significantly prevent the short or long terms neurotoxic effects of OPNA exposure (Doctor and Saxena, 2005; Rahimi et al., 2006). Recently FDA-approved midazolam worked better in reducing the seizurogenic effects of OPNA when administered within 20 min of exposure (McDonough et al., 1999; Leikin et al., 2002; Chapman et al., 2015). The initial SE severity determines the extent of brain injury and the progression of epileptogenesis, characterized by SRS. Our previous studies have demonstrated that by modifying the early epileptogenic events such as preventing neuroinflammation and neurodegeneration, we could significantly alter the progression of the disease (Puttachary et al., 2016; Gage et al., 2022).

Several other approaches have also demonstrated that early intervention post-SE can yield better outcomes. For example, cannabinoids have been widely used for the initial screening as antiepileptogenic drug in pilocarpine and kainic acid epilepsy models (Marsicano et al., 2003). Drugs to block IL-1R1/TLR4 pathway have been shown to interfere with the mechanisms of epileptogenesis (Iori et al., 2017). Rapamycin that targets mTOR pathway (Zeng et al., 2009), COX-2 inhibitors (Rojas et al., 2014), JAK–STAT inhibitors (Grabenstatter et al., 2014) and neurosteroids (Biagini et al., 2010) have been investigated. In our previous studies, we identified the Src/Fyn tyrosine kinase and iNOS as potential disease-modifying targets in the DFP and kainate models of epilepsy (Puttachary et al., 2016; Sharma et al., 2018; Putra et al., 2020; Sharma et al., 2021). The present study is a logical extension of our nitrooxidative stress modulating approach with a focus on dose optimization of 1400W. The nitrooxidative stress in epileptogenesis has emerged as a potential therapeutic target in temporal lobe epilepsy models (Puttachary et al., 2015; Maes et al., 2020; Gage et al., 2022). Nitrooxidative stress is a bi-product of gliosis and neuroinflammatory process which mediate neurodegeneration pathways following SE (Shakeel et al., 2017; Gage et al., 2022). SE induced damage has been directly correlated to a higher level of ROS/RNS in the brain (Patel, 2016; Pearson-Smith and Patel, 2017; Terrone et al., 2020). NOS catalyzes the production of NO, which is an important cellular signaling molecule and is highly reactive. It reacts with superoxide radicals to form various RNS (Squadrito and Pryor, 1998; Droge, 2002). RNS are mainly antimicrobial in nature and are produced by immune cells which also express iNOS in response to exposure to infection or trauma in other organs. We have recently shown a significant increase in iNOS and 3-NT production in the brain after exposure to DFP (Putra et al., 2020). Therefore, iNOS inhibitors can dampen SE-induced cellular changes in the brain.

Previously we tested 1400W, a selective iNOS inhibitor, in kainic acid and DFP models of epilepsy. In these studies, 1400W was used at 20 mg/kg, given for 3 days at 12 h intervals starting ~4 h after exposure to kainic acid or DFP. The aim was to investigating the long-term (3 months in the rat DFP model and 6 months in the rat KA model) effects of 1400W in modifying the SE-induced epileptogenesis (Puttachary et al., 2016; Putra et al., 2020). In the DFP model, the treatment regimen was effective in those animals that had mild to moderate SE severity. Therefore, in this study, we tested the neuroprotective and anti-nitrooxidative efficacy of 1400W when it was administered twice daily for the first 3 days followed by single dose for 4 or 11 days in the rat DFP model. SE severity determines the onset of epileptogenesis and the disease progression. Therefore, in an experimental design for disease-modifying effects of a test drug, balancing the initial SE severity between the vehicle and test drug groups is critical. A rigorous SE severity quantification and SE severity balance between the vehicle and test drug groups will mitigate experimental bias and the confounding effects of the test drug. We have recently demonstrated the differential impact of SE severity on epileptogenesis and behavioral outcome (Gage et al., 2020, 2021). In this study, we randomly distributed animals from each sex to either vehicle or 1400W treatment. The mean SE severity was very similar with no significant differences between groups (Figure 2; Table 1). Therefore, the findings in this study are the true effects of 1400W.

It has been demonstrated that DFP treatment in the rat model can induce significant gliosis and neurodegeneration at 7 day post exposure (Liu et al., 2012; Flannery et al., 2016; Gage et al., 2021, 2022). In this study, we observed a significant increase in microglia and astroglia across various brain regions at 8 and 15-days post DFP exposure, when compared to control animals (Figures 3, 4). 1400W at 10 mg/kg for a week or 2 weeks was effective in reducing astrogliosis in all brain regions, but one-week treatment was not effective on microgliosis. This is likely due to increased number of brain infiltrated peripheral leukocytes or monocytes that may have later differentiated into microglia during the first week of post-SE (Varvel et al., 2016; Baufeld et al., 2018; Bosco et al., 2020) However, 15 mg/kg dose of 1400W for a week was effective in mitigating the DFP-induced microgliosis. There were no sex differences in the efficacy of 1400W at either concentration or the duration of treatment (Supplementary Figures S1, S2).

The clinical and experimental data that suggest that neurodegenerative diseases and acquired epilepsy share similar pathways (Casillas-Espinosa et al., 2020). Alzheimer’s disease patients can develop epilepsy as a comorbidity (Abou-Khalil, 2010; Friedman et al., 2012) and seizures can worsen the progression of dementia due to enhanced neurodegeneration [Volicer et al., 1995; Federal Insecticide, Fungicide and Rodenticide Act (FIFRA), 2020]. Thus, a pharmacological intervention that targets the neurodegenerative processes may potentially be anti-epileptogenic and can exert a disease modifying effect post SE. In this study, a significant rise in degenerating neurons were seen in all brain regions following DFP exposure at 8 and 15-day post DFP compared to control (Figures 5, 6). 1400W at 15 mg/kg was more effective in reducing neurodegeneration both for 1 week and two-week treatment, whereas 10 mg/kg was effective when given for two-weeks. The 1400W-mediated neuroprotective effect was comparable with reduction in microgliosis and nitrooxidative stress. The microglia-mediated neuronal injury could be due to a significant lipid peroxidation of neuronal membranes and modification of structural proteins and nucleic acid by ROS/RNS (Cobley et al., 2018; Borowicz-Reutt and Czuczwar, 2020).

Activated glial cells are known to produce pro-inflammatory cytokines in the serum, a hallmark of neuroinflammation (De Simoni et al., 2000; Jankowsky and Patterson, 2001; Putra et al., 2020; Sharma et al., 2021). Activated immune cells and glia also upregulate iNOS which can further mediate the release of pro-inflammatory cytokines through NFκB signalling (Chang et al., 2014). Thus, iNOS is a potential target for disease modifying strategy in OP induced SE. At 8-day and 15-day post DFP exposure, we found a significant increase in serum IL-1β, IL-6, TNF-α and MCP-1. 1400W treatment did reduce the levels of the pro-inflammatory cytokines but had a dose dependent and time dependent effect (Figure 7). Previous research has shown that IL-1β contributes to epileptogenesis by altering transcription pathways and ion channel function and can ultimately affects the neuronal survival (Allan et al., 2005; Vezzani and Baram, 2007). Increased IL-6 production has been showed in patients with refractory epilepsy and experimental models of temporal lobe epilepsy (Liimatainen et al., 2009), where in an early rise in IL-6 levels were correlated with acute epileptic seizures onset (Alapirtti et al., 2018). TNF-α is considered to be the master regulator of pro-inflammatory cytokines and is secreted by reactive microglia and reactive astroglia. TNF- α regulates the neuronal activity by directly affecting the release of glutamate or γ-aminobutyric acid (GABA; Chen et al., 2021). MCP-1 which was also upregulated in DFP exposed animals and mitigated by 1400W in this study, as in our previous study (Putra et al., 2020) implies that the results are reproducible. Reactive astrocytes are known to produce MCPI which acts on CCR2 receptors expressed by stressed neurons and reactive microglia. This chemotaxis mechanism has been known to induce neurodegeneration *via* STAT3 pathway after SE in a mouse model (Arisi et al., 2015; Tian et al., 2017). Significant suppression of pro-inflammatory cytokines in this study supports the claim that 1400W with an optimized dosing regimen can be neuroprotective.

Peripheral nitrooxidative stress biomarkers of epileptogenesis such as nitrite, ROS, and oxidized glutathione are the by-products of reactive gliosis, and reliable readouts for evaluating the efficacy of disease-modifiers. Several experimental models of epilepsy demonstrate the central role of oxidative stress in epileptogenesis (Puttachary et al., 2015; Rowley et al., 2015). ROS/RNS have been shown to oxidize neurotransmitter, impair cellular functions, damage mitochondrial DNA, cause lipid peroxidation of neuronal membranes and ultimately lead to neurodegeneration with reduced seizure threshold (Cobley et al., 2018; Borowicz-Reutt and Czuczwar, 2020). In our study, we observed elevated levels of ROS and RNS in serum 8-day and 15-day post DFP suggesting their neurotoxic effects. 1400W therapy at 15 mg/kg supressed the ROS/RNS production demonstrating its potential as a disease modifying agent in an acute model of DFP induced neurotoxicity (Figure 9).

The majority of the anti-epileptics are symptomatic. Therefore, a combination therapy with a promising antioxidant may enhance the efficacy of antiseizure medication. Successful promotion of endogenous antioxidant system by activating nuclear factor erythroid 2 -related factor 2 (Nrf2) led to 94% reduction in late spontaneous seizures onset (Shekh-Ahmad et al., 2018). The most significant non-enzymatic oxidant defense in the body is glutathione (GSH), where the levels of GSH and oxidized glutathione (GSSG) serve as a biomarker for oxidative stress in the body. In our study, a significant rise in GSSG and lower levels of GSH following DFP at 8 and 15-day post DFP suggested an upregulated oxidative state. 1400W at 15 mg/kg for 2 weeks was able to lower the GSSG levels with a concomitant rise in GSH (Figure 8). This shows that 1400W not only acted directly to inhibit iNOS but also exerted modulatory effect on the endogenous antioxidant system. Other exogenous antioxidants such as N-acetylcysteine and sulforaphane has been previously shown to potentiate the glutathione antioxidant system which controlled the oxidative stress in animal models and modified epileptogenesis (Liang and Patel, 2006; Carrasco-Pozo et al., 2015; Pauletti et al., 2019; Mohammadi et al., 2022). A seizure initiates, when a sufficient number of neurons depolarize and produce action potentials (Holmes and Ben-Ari, 2001). An insult or injury to a healthy brain can induce SE which can further progress into SRS. vEEG plays a key role in rigorous monitoring of SRS that records electrographic seizures and epileptiform spikes activity. The association of prolonged SRS with other comorbid conditions like diabetes (Wu et al., 2018), respiratory disorders (Tang et al., 2014), depression, anxiety (Kanner, 2011; Tao and Wang, 2016), and cognitive impairments (Cretin, 2018) can be a real cause of concern. Early administration of diazepam within 10 min (Todorovic et al., 2012) or midazolam within 20 min (Wu et al., 2018) reduces SE severity, protect the brain, and may prevent or delay SRS. We have previously demonstrated that how a prolonged SE and its mitigation in a timely manner had significant effect on reducing the brain pathology (Gage et al., 2021). Also, we have demonstrated the sex differences in SE severity and onset of SRS can also play a significant role in testing of any disease modifying therapy (Gage et al., 2020, 2022; Rao et al., 2022). Several AEDs have aided in the reduction of SRS, but generally the proportion of drug resistant patients with epilepsy have not significantly changed (Pellock, 2000; Luciano and Shorvon, 2007; Gilioli et al., 2012; Janmohamed et al., 2020). In this study, we analyzed the electrographic seizure and electrical spiking activity in the brain up to 15-days post DFP and did not observe any significant changes with 1400W administration in the DFP exposed animals (Figure 10). We have noticed a similar trend in our previous studies, where we did not observe reduction of seizure and spiking activity in less than 4 weeks of treatment (Putra et al., 2020). This could be explained by the fact that this is too early for 1400W to influence the electrical activity of the brain.

In summary, DFP exposure induced neuroinflammatory response in the brain and serum which was (Capacio et al., 2004) evident from a robust gliosis in different regions of the brain and increased proinflammatory cytokines in the serum. Increased neurodegeneration at both 8 and 15-days in several brain regions suggests the impact of SE severity, induced by the DFP exposure. Elevated pro-inflammatory cytokine levels, increased glutathione oxidation, and elevated ROS and RNS levels following DFP exposure suggests the DFP-induced brain injury and its impact on the peripheral biomarkers. 1400W was partially effective with 10 mg/kg for a week regimen in reducing the gliosis, neurodegeneration and some neuroinflammatory biomarkers. A higher concentration of 15 mg/kg 1400W for 2 weeks was effective on all DFP-induced parameters despite severe SE in both sexes. We also found that short-term telemetry studies immediately post-exposure or during the treatment regimen do not yield meaningful outcomes while the brain is undergoing reorganization in response to an insult or a reversal drug.

## Data availability statement

The original contributions presented in the study are included in the article/Supplementary material, further inquiries can be directed to the corresponding author.

## Ethics statement

The animal study was reviewed and approved by the Iowa State University IACUC.

## Author contributions

TT secured funding for the project, designed experiments, verified the data, offered intellectual input, and edited the manuscript. NM, SS, MS, MG, NR, and CM conducted the experiments, compiled and crossverified the data, proofread the manuscript with a significant contribution from NM and SS. MG, NM and SS acquired, and analyzed the EEG data and graphed the data. NM pooled the other data, cross verified, and graphed the data. All authors contributed to the article and approved the submitted version.

## Funding

The Principal Investigator (TT) was received the funding for this project from the National Institutes of Health/NINDS (U01 NS117284).

## Conflict of interest

The authors declare that the research was conducted in the absence of any commercial or financial relationships and none of the authors have any conflict of interest.

## Publisher’s note

All claims expressed in this article are solely those of the authors and do not necessarily represent those of their affiliated organizations, or those of the publisher, the editors and the reviewers. Any product that may be evaluated in this article, or claim that may be made by its manufacturer, is not guaranteed or endorsed by the publisher.
