# Supplementary figures and images for: 1400 W, a selective inducible nitric oxide synthase inhibitor, mitigates early neuroinflammation and nitrooxidative stress in diisopropylfluorophosphate-induced short-term neurotoxicity rat model
Source: Front Mol Neurosci. 2023 Mar 17;16:1125934. doi: 10.3389/fnmol.2023.1125934 (PMC10064070; doi:10.3389/fnmol.2023.1125934)

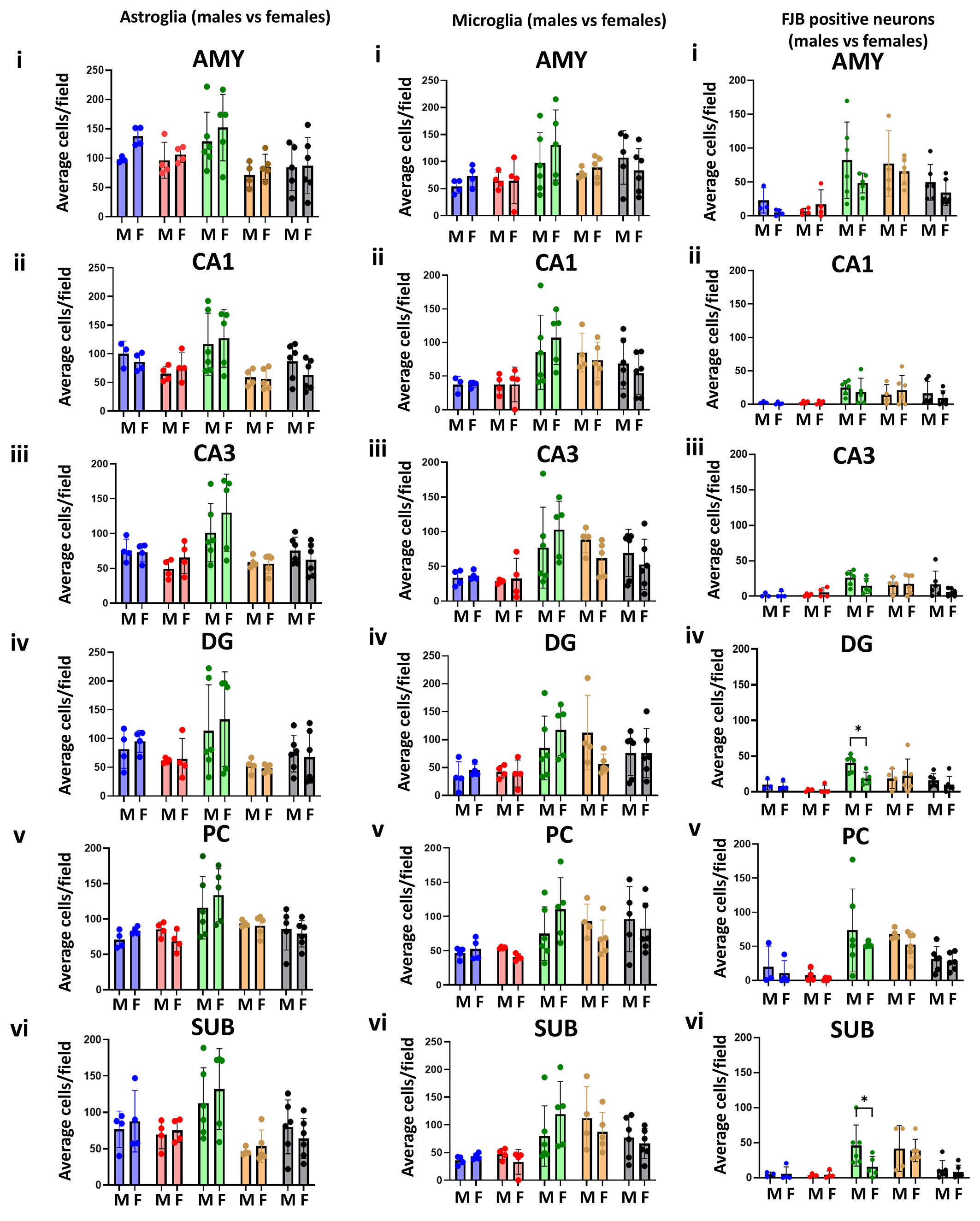

Supplement: Supplementary file 1 [file image_1.tif]

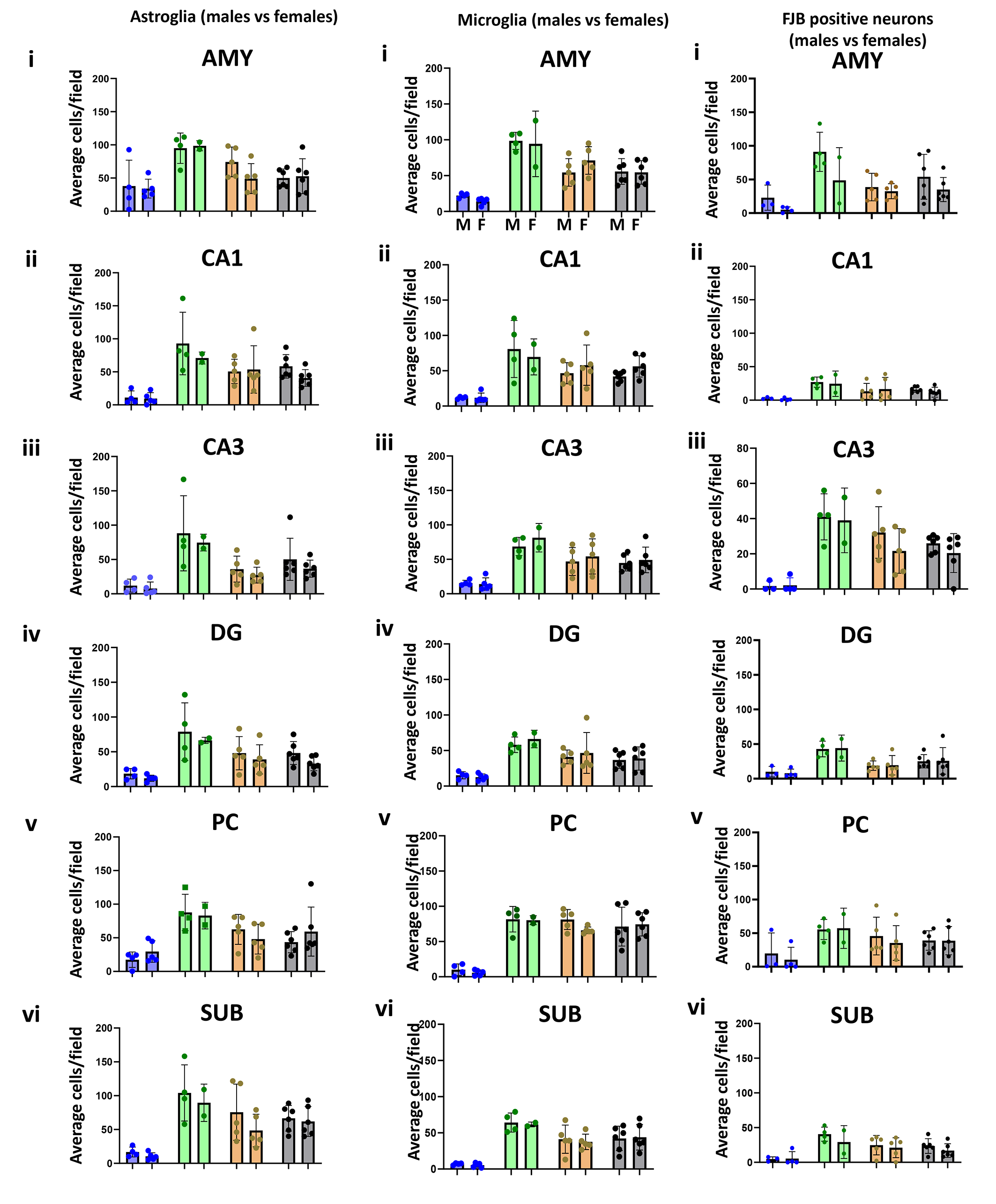

Supplement: Supplementary file 2 [file image_2.tif]

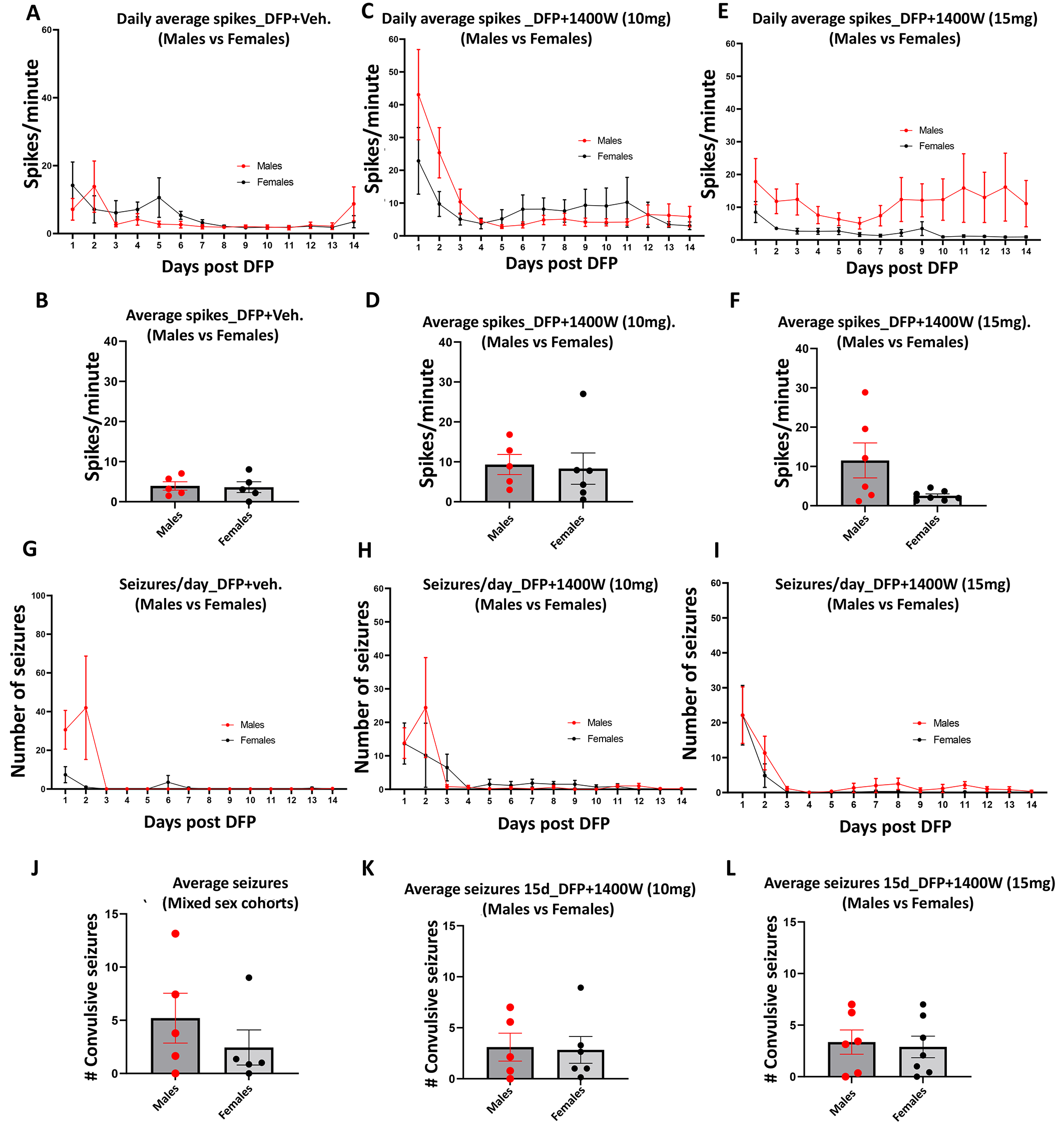

Supplement: Supplementary file 3 [file image_3.tif]
